# Alpha-Thalassemia Unmasked in a Patient With Sickle Cell Trait: A Case Report

**DOI:** 10.7759/cureus.92353

**Published:** 2025-09-15

**Authors:** Bryan Chuquimarca, Cinthya Carrión, Victoria Matosas, Eloisa Riva

**Affiliations:** 1 Hematology, Hospital de Clínicas, Montevideo, URY; 2 Faculty of Medical Sciences, Universidad Central del Ecuador, Quito, ECU

**Keywords:** alpha-thalassemia, gap-pcr, genetic counseling, hemoglobinopathy, microcytic anemia, ntdt, sickle cell trait

## Abstract

Sickle cell trait is usually an asymptomatic, benign carrier state with mild or no anemia. We present the case of a 59-year-old female with sickle cell trait and mild persistent microcytic anemia. Iron studies were normal, and ferritin was elevated. Peripheral smear demonstrated microcytosis and occasional sickled cells. Given the marked microcytosis, molecular analysis was performed and confirmed homozygous alpha-thalassemia via gap-polymerase chain reaction. The coexistence of sickle cell trait and homozygous alpha-thalassemia accounted for the hematologic findings. Recognition of this genotype is critical to prevent misdiagnosis, avoid unnecessary iron supplementation, and guide appropriate genetic counseling and follow-up. Co-inheritance of hemoglobinopathies may present diagnostic challenges and require further investigation, particularly when laboratory results are inconsistent with expected phenotypes. In cases of unexplained microcytosis, especially in individuals with known hemoglobin variants, molecular testing for alpha-thalassemia should be considered to ensure accurate diagnosis and tailored management.

## Introduction

Sickle cell trait (HbAS) is usually a benign carrier state resulting from heterozygosity for the *β-globin* gene mutation responsible for hemoglobin S (HbS), with a prevalence of approximately 15.5 per 1,000 live births in the United States, and higher rates in populations of African, Mediterranean, Middle Eastern, and South Asian ancestry [1]. Although typically asymptomatic, sickle cell trait may lead to diagnostic challenges when co-inherited with other hemoglobinopathies, such as alpha-thalassemia, which affect red blood cell indices and hemoglobin composition [2]. Alpha-thalassemia is among the most common monogenic disorders worldwide, caused by deletions in one or more *α-globin* genes [3].

The clinical overlap between sickle cell trait and alpha-thalassemia trait can obscure diagnosis, mislead treatment, and contribute to iron overload due to ineffective erythropoiesis or inappropriate iron supplementation. Laboratory tests often show persistent microcytosis, anemia, and lower HbS levels [2-4]. In HbAS carriers, HbS typically ranges between 35% and 45%, but the presence of alpha-thalassemia may reduce this proportion due to decreased availability of α-chains, with levels between 25% and 35% often indicating homozygous alpha-thalassemia trait (-α/-α) or heterozygous deletion (-/αα) [2]. This genotype interaction not only impacts phenotypic presentation but also has reproductive implications for offspring and the need for appropriate genetic counseling [5].

Molecular testing methods such as gap-polymerase chain reaction (PCR) are essential in confirming alpha-thalassemia genotypes and should be considered when microcytosis persists despite normal iron studies or when the hemoglobin electrophoresis pattern does not align with typical clinical findings [2,6]. Recognition of compound heterozygosity for HbAS and alpha-thalassemia is crucial for guiding appropriate clinical monitoring, reproductive counseling, and avoiding unnecessary interventions [5].

We present the case of a 59-year-old female previously diagnosed with sickle cell trait, who presented with persistent microcytic anemia unresponsive to iron supplementation. The degree of anemia, marked microcytosis, and atypically low HbS percentage on hemoglobin electrophoresis could not be explained by sickle cell trait alone, raising suspicion for an additional underlying hemoglobinopathy. Subsequent molecular testing confirmed homozygous -α³·⁷/-α³·⁷ alpha-thalassemia. This diagnostic step not only clarified the etiology of her anemia but also changed the long-term clinical management and follow-up strategy, underscoring the value of considering treatment failure and laboratory inconsistencies as key triggers for expanding the diagnostic workup.

This article was previously presented as an award-winning oral presentation at the XVII Uruguayan Congress of Hematology, held on September 16th, 2023.

## Case presentation

A 59-year-old woman with a prior diagnosis of sickle cell trait without chronic complications was referred for evaluation of persistent microcytic anemia, which was being treated with iron supplementation without improvement for six months. She reported chronic mild fatigue but denied pallor, bleeding, or dietary deficiencies. Physical examination revealed an enlarged spleen. Family history was notable for chronic anemia in siblings and parental relatives.

Laboratory evaluation revealed a hemoglobin level of 8.3 g/dL, with marked microcytosis (mean corpuscular volume of 69.5 fL) and a corpuscular mean hemoglobin concentration of 28 g/dL. Iron studies showed elevated ferritin (523 ng/mL) and a normal transferrin saturation of 23%, ruling out iron deficiency (Table 1).

**Table 1:** Hematological and iron studies showing microcytic, hypochromic anemia in the setting of normal iron stores.

Test	Value	Reference range and units
Hemoglobin	8.3	12.0–15.5 g/dL
Mean corpuscular volume	69.5	80–100 fL
Corpuscular mean hemoglobin	28	32.0–36.0 g/dL
Ferritin	523	12–150 ng/mL
Transferrin saturation	23	20–50%

**Figure 1:**
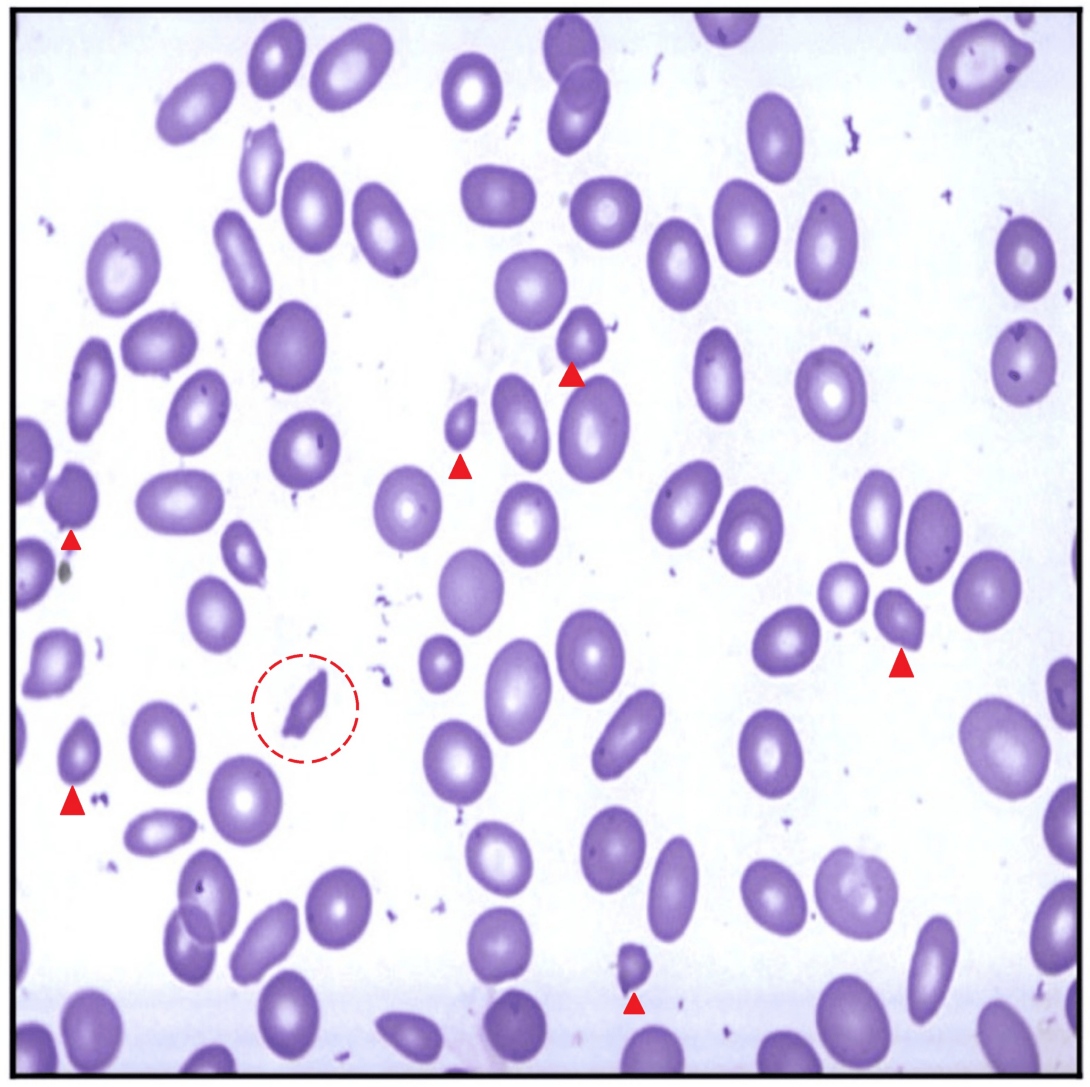
Peripheral blood smear (May-Grünwald Giemsa stain, 100× oil immersion). The image shows red blood cells with marked microcytosis (arrowheads) and occasional sickled erythrocytes (open circle). No nucleated red blood cells were observed.

The peripheral blood smear demonstrated microcytosis, target cells, and occasional sickled erythrocytes (Figure 1). Capillary electrophoresis revealed HbA at 70%, HbS at 26.8%, and HbA2 at 3.2% (Table 2), consistent with sickle cell trait. However, the patient’s HbS level was lower than expected for sickle trait, and in the context of significant microcytosis without iron deficiency, this finding raised suspicion of coexisting alpha-thalassemia.

**Table 2:** Hemoglobin capillary electrophoresis consistent with sickle cell trait, with low hemoglobin S value.

Hemoglobin fraction	Percentage
Hemoglobin A	70%
Hemoglobin S	26.8%
Hemoglobin A2	3.2%

**Figure 2:**
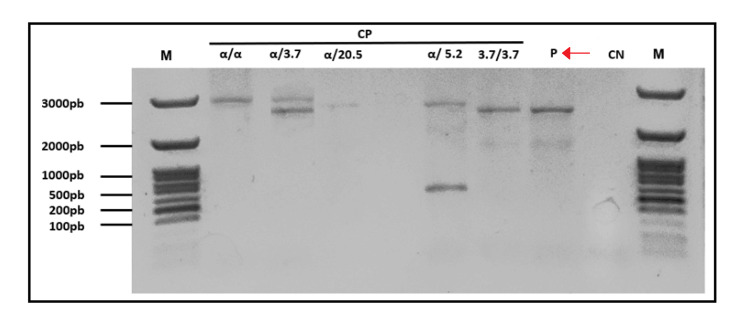
Gap-PCR multiplex for alpha-thalassemia deletion screening compatible with homozygous alpha-thalassemia. The patient lane (arrow) shows a single band corresponding to the size of a known positive control with a homozygous -α³·⁷/-α³·⁷ deletion (3.7/3.7), confirming the diagnosis of homozygous α⁺-thalassemia. In this assay, the presence or absence of specific amplified fragments allows the differentiation of normal alleles from common *α-globin* gene deletions. A normal control (α/α) displays only the expected wild-type band, while CPs with known genotypes exhibit characteristic bands for each deletion: heterozygous -α³·⁷ (α/3.7), heterozygous -α⁵·² (α/5.2), and heterozygous -α²⁰·⁵ (α/20.5). The patient’s result, showing only the homozygous -α³·⁷/-α³·⁷ deletion band without the normal allele band, is consistent with the homozygous state. A CN ensures no contamination, and M on both sides of the gel confirm the fragment sizes for accurate interpretation. M: molecular size markers; pb: base pair length of PCR fragments; P: patient; CP: positive controls; CN: negative control; PCR: polymerase chain reaction

We conducted a gap-PCR for common *α-globin* gene deletions. This technique amplifies specific genomic regions surrounding known deletion breakpoints, allowing differentiation between normal alleles and the most frequent alpha-thalassemia deletions. Gap-PCR analysis demonstrated that the patient’s sample presented a single amplification band at the expected size for the homozygous -α³·⁷ deletion, and no band corresponding to the intact *α-globin* gene, consistent with a homozygous -α³·⁷/-α³·⁷ genotype (Figure 2).

These results confirmed the coexistence of sickle cell trait with homozygous α⁺-thalassemia, explaining the patient’s disproportionate microcytosis, the lower HbS proportion, and the absence of response to iron therapy. The patient was reassured and educated on her carrier status for both conditions. Iron supplementation was avoided. Genetic counseling was offered, particularly due to potential reproductive implications in her relatives, and family testing was indicated.

## Discussion

The coexistence of sickle cell trait and alpha-thalassemia is not uncommon but frequently underrecognized in areas with low incidence. Both conditions may independently cause red cell abnormalities and mild anemia, and concurrence is relatively common in malaria-endemic areas, but concurrence frequency varies by region and population [7].

Alpha-thalassemia can be misclassified as iron-deficiency anemia, potentially leading to inappropriate supplementation. Diverse genetic profiles lead to unique anemia degrees and levels of HbS, with homozygous states being significantly symptomatic. Furthermore, accurate identification has relevance for genetic counseling and understanding potential complications of non-transfusion-dependent thalassemia (NTDT) [2,8,9]. This case underlines the importance of considering co-inherited hemoglobinopathies and utilizing molecular tools to achieve diagnostic accuracy.

This case highlights the diagnostic complexity in evaluating microcytic anemia when co-inherited hemoglobinopathies are present. The patient exhibited mild anemia with microcytosis, alongside a peripheral smear showing occasional sickle cells. Capillary hemoglobin electrophoresis revealed 70% HbA, 26.8% HbS, and 3.2% HbA2, consistent with sickle cell trait. However, the degree of microcytosis (mean corpuscular volume of 69.5 fL) and HbS fraction was disproportionate to what is typically observed in sickle cell trait alone, prompting further investigation.

Gap-PCR confirmed a homozygous -α³·⁷ deletion (-α³·⁷/-α³·⁷), establishing a diagnosis of homozygous alpha⁺-thalassemia. This condition is generally asymptomatic and results in mild microcytic anemia due to reduced α-globin chain production. Importantly, this finding explains the hematologic abnormalities without evidence of iron deficiency, as iron studies were normal and ferritin levels were elevated (523 ng/mL), likely from ineffective erythropoiesis.

Although homozygous -α³·⁷ thalassemia is typically classified as a benign trait, it falls under the broader category of NTDT, for which the Thalassaemia International Federation (TIF) guidelines emphasize that even mildly anemic NTDT patients can experience long-term complications due to ineffective erythropoiesis and iron metabolism [8,9].

TIF guidelines recommend periodic monitoring of hemoglobin levels, iron status, and organ function. In this patient, routine transfusion was not indicated; however, the elevated ferritin (523 ng/mL) warranted closer follow-up. Ferritin should be reassessed every three to six months, and liver iron concentration (LIC) by MRI should be considered if levels persist above 500-800 ng/mL. Iron chelation therapy is generally not required unless LIC exceeds 5 mg/g dry weight or ferritin remains persistently above 800 ng/mL [9]. Bone health should also be monitored with a dual-energy X-ray absorptiometry scan every two to three years, given the risk of osteopenia in NTDT patients. Although rare in homozygous -α³·⁷ individuals, extramedullary hematopoiesis and vascular complications (e.g., pulmonary hypertension, thrombosis) have been described in NTDT populations and should be evaluated clinically if symptoms arise [3,9].

Reproductive genetic counseling and family study are critical. As a homozygous -α³·⁷ carrier, the patient’s children are obligate heterozygotes for alpha-thalassemia, reinforcing the need for family counseling to reduce offspring with HbH disease or more severe phenotypes [5,9].

## Conclusions

Microcytic anemia in patients with sickle cell trait should not be automatically attributed to iron deficiency, and no response to treatment should raise clinical concern of an alternative diagnosis. This case illustrates the diagnostic importance of considering co-inherited hemoglobinopathies in sickle cell trait patients with microcytic anemia and normal iron studies. These signs led to further investigation and the identification of homozygous alpha-thalassemia through molecular testing. While both conditions are typically benign in isolation, their coexistence can influence laboratory findings, clinical interpretation, and long-term follow-up. Appropriate diagnosis prevents unnecessary treatments such as iron supplementation and enables tailored counseling regarding genetic risks and future reproductive planning. Incorporating NTDT monitoring guidelines from the TIF may be beneficial even in mildly affected patients to guide surveillance of iron burden, bone health, and potential chronic complications.

## References

[1] Brousseau DC, Panepinto JA, Nimmer M, Hoffmann RG (2010). The number of people with sickle-cell disease in the United States: national and state estimates. Am J Hematol.

[2] Giordano PC (2013). Strategies for basic laboratory diagnostics of the hemoglobinopathies in multi-ethnic societies: interpretation of results and pitfalls. Int J Lab Hematol.

[3] Piel FB, Weatherall DJ (2014). The α-thalassemias. N Engl J Med.

[4] Raffield LM, Ulirsch JC, Naik RP (2018). Common α-globin variants modify hematologic and other clinical phenotypes in sickle cell trait and disease. PLoS Genet.

[5] Pinto VM, De Franceschi L, Gianesin B (2023). Management of the sickle cell trait: an opinion by expert panel members. J Clin Med.

[6] Chong SS, Boehm CD, Higgs DR, Cutting GR (2000). Single-tube multiplex-PCR screen for common deletional determinants of alpha-thalassemia. Blood.

[7] Santos B, Delgadinho M, Ferreira J (2020). Co-Inheritance of alpha-thalassemia and sickle cell disease in a cohort of Angolan pediatric patients. Mol Biol Rep.

[8] Musallam KM (2013). Iron overload in non-transfusion-dependent thalassemia. Thalass Rep.

[9] Taher A, Musallam K, Cappellini MD (2017). Guidelines for the Management of Non Transfusion Dependent Thalassaemia (NTDT). Nicosia, Cyprus: Thalassaemia International Federation.

